# National priorities for oral health research, Peru 2022-2026: process, experiences and perspectives

**DOI:** 10.17843/rpmesp.2023.403.12082

**Published:** 2023-09-28

**Authors:** Adriana Echevarria-Goche, Gilmer Solis-Sánchez, Lesly V. Tuesta-Orbe, Christian Andamayo-Flores, Margot Vidal-Anzardo

**Affiliations:** 1 Instituto Nacional de Salud, Lima, Peru. Instituto Nacional de Salud Lima Peru; 2 Ministerio de Salud, Lima, Peru. Ministerio de Salud Lima Peru

**Keywords:** Research Priorities, Public Health Systems Research, Community-Based Participatory Research, National Health Policy, Health Research Policy, Health Priorities, Dental Research, Oral Health, Peru

## Abstract

The National Institute of Health has, for the first time, identified National Priorities for Oral Health Research, this process was carried out by the Subdirectorate of Research and Laboratories of Noncommunicable Diseases of the National Center for Public Health with the technical advice of the Directorate of Research and Innovation in Health and in coordination with the Executive Directorate of Oral Health of the General Directorate of Strategic Interventions in Public Health of the Ministry of Health, using a participatory methodology with three key actors: researchers/specialists, experts and decision-makers. This article aims to describe the process used to identify these priorities, which consisted of five phases: i) identification of the strategic objectives of the Ministry of Health, ii) identification of needs in oral health research, iii) review by experts and assessment of needs according to criteria and v) presentation of the priorities. As a result, 12 priorities were obtained, which were subsequently approved by Ministerial Resolution No. 262-2022/MINSA, for a period of 2022-2026. In addition, we provide recommendations for future processes.

## INTRODUCTION

Article 14 of the Political Constitution of Peru states “to promote the scientific and technological development of the country” as a duty of the State ^(^^1^. No. 26842, General Health Law, establishes that “the State promotes scientific and technological research in the field of health, as well as the education, teaching and training of human resources for health care” ^2^; from which it can be interpreted that the advancement of science derives from education and research, which are the pillars for the development of society, and the State guarantees their promotion.

The Ministry of Health (MINSA), as an agency of the executive branch of the government ^3^, has the mission of “protecting personal dignity, promoting health, preventing diseases and guaranteeing comprehensive health care for all the inhabitants of the country, proposing and conducting health policy guidelines in consultation with all public sectors and social actors” ^4^. The incorporation of health research and technology under the scope of the competence of MINSA has been established since 2013 with the publication of Legislative Decree No. 1161, which approves the Law on the Organization and Functions of MINSA, ^5^.

Likewise, the vision of the National Institute of Health (INS) is “to be a modern, dynamic and leading institution at the national and international level in the generation, development and transfer of technologies and scientific knowledge in biomedical research [... ]” ^6^. Within this framework, the INS leads the development of the process for the identification of research priorities at the national level, with the objective of generating knowledge in the field of public health in order to guide the formulation, implementation and evaluation of policies and interventions that allocate resources for the attention of prioritized health problems due to the fact that resources in Peru are limited ^7^.

The purpose of this article is to describe the process followed by the Subdirectorate of Research and Laboratories for Noncommunicable Diseases (SUDENT) of the National Center for Public Health (CNSP), with the technical advice of the Directorate of Research and Innovation in Health (DIIS) and in coordination with the Executive Directorate of Oral Health of the General Directorate of Strategic Interventions in Public Health (DGIESP) of MINSA, for the identification of “National Priorities for Research in Oral Health”; as well as to provide recommendations for future processes.

## PREVIOUS PROCESSES

Historically, experiences in Peru have been developed since 1974 to identify health research priorities, each of which has guided health policies since then. In the first process, MINSA expressed the intention to support health institutes for research. The National Action Plan was developed during the second process (1983-1984) aimed at achieving “health for all by the year 2000”. During the third process (1991-1994), the National Council for Science, Technology and Innovation (CONCYTEC) and the Pan American Health Organization (PAHO) worked on four priority topics and channeled available resources. In the fourth process (1996-1997), the MINSA Health Research Policy Commission identified new thematic areas and prepared 234 study sheets. During the fifth process (2001-2002), the INS defined the research guidelines through an internal seminar-workshop. Finally, during the sixth process (2006-2007), according to Cabezas *et al*. ^8^, quoted by the Directorate of Health Research and Innovation ^9^, “the INS deepens the scope and methodology of establishing research priorities” consolidating its participatory methodology.

The process of identification of regional priorities was carried out for the first time in 2019, which established the priorities for the 2010-2014 period, defining “health research” as: “the production of knowledge aimed at studying the health conditions (at the biological, psychological and social levels) of an individual or population and the responses of society to improve them” ^8^.

The process that started in 2009 continued during 2014 with the development of workshops in the 24 regions of Peru and Metropolitan Lima with the participation of regional health authorities, public and private universities, public and private research entities, and other regional governmental sectors convened by the INS to establish the National Research Priorities 2016-2021^7^^,^^9^. These priorities were implemented in 2019, through the Ministerial Resolution No. 658-2019/MINSA, which approves the National Research Priorities in Health in Peru 2019-2023, identifying the following health problems: (i) Traffic accidents; (ii) Cancer; (iii) Metabolic and cardiovascular diseases; (iv) Respiratory infections and pneumonia; (v) Sexually transmitted infections and HIV-AIDS; (vi) Malnutrition and anemia; (vii) Metaxenic and zoonotic diseases; (viii) Environmental and occupational health; (ix) Maternal, perinatal and neonatal health; and (x) Mental health ^10^. It is important to mention that, as stated in the document of the identification process of these National Health Research Priorities, one of the health problems identified was “oral health” ^11^; however, since the problems that obtained the highest scores were prioritized, oral health did not appear in the final prioritization list.

In this scenario, oral health requires a revaluation in order to gain greater attention regarding research in our country after having been relegated for decades, justifying a specific identification of Oral Health Research Priorities.

In 2021, the National Institute of Health, through SUDENT of the CNSP, incorporated oral health as an area of work, establishing the “Oral Health Plan 2021-2023”, which began with the identification of National Oral Health Research Priorities 2021-2025, and whose process will be described in this article ^12^. This prioritization will be fundamental to guide the development of cost-effective research and to optimize the use of the country’s economic resources, taking the previous process as a reference ^7^.

## THE PROCESS

The identification of Oral Health Research Priorities, which took place from June 2 to June 25, 2021, was led by the SUDENT technical team, which was made up of four professionals (one physician and three dental surgeons) who had experience in research and academic training in fields related to Public Health, Epidemiology and/or Health Services Management. The DIIS technical team (the office that has led the National Research Priorities process and validated the methodology during the application of this research prioritization process) also provided advice, and worked in close coordination with the technical team of the Executive Directorate of Oral Health (DSABU) of the DGIESP of MINSA. The activities were developed through virtual workshops via the Zoom platform based on the participatory methodology adopted from the “Guide for the identification of Regional Health Research Priorities” prepared by the DIIS of the INS ^9^^,^^13^.

Three key actors were identified based on the following characteristics (Table 1):

1. Researchers/specialists, who met at least two of the four conditions: i) being a researcher recognized by the National Scientific, Technological and Technological Innovation Registry (RENACYT) (via DINA-CONCYTEC platform ^14^ at the date of download as of March 30, 2021; ii) having published two or more research articles with relevance in oral health (letters to the editor were not considered) in national or international peer-reviewed journals; iii) teaching in a public or private university; and iv) being a specialist in a dental area. The SUDENT technical team was able to access the telephone numbers and/or email addresses of 78 professionals from the list of identified professionals who met the above conditions, which were used to contact them through social networks, telephone calls and/or WhatsApp in order to ask them to participate in the process; of the 78 contacted professionals, 46 (59.0%) responded and accepted. This call took place between May 18 and 28, 2021.

2. Decision-makers, made up of the three members of the team of the Executive Directorate of Oral Health (due to availability in some phases 2 were involved) and the 29 Coordinators of the oral health program belonging to the Regional Health Directorates (DIRESAS), Regional Health Managements (GERESAS) and Integrated Health Networks Directorate (DIRIS). The list of coordinators of the national oral health program was provided by the DSABU, which provided logistical support to manage the call via email; this call took place between May 18 and 28, 2021. All coordinators responded and accepted, for a total of 32 decision makers.

3. Experts in Oral Health, formed by *ad honorem* members of the Committee of Experts of the DSABU of the DGIESP through Ministerial Resolution No. 571-2017/MINSA ^15^. DSABU provided the contacts of the seven members of the referred committee, who were summoned by phone calls and/or WhatsApp between May 18 and May 28, 2021. After the call, three experts (42.9%) responded and accepted the invitation. 

The phases of the process are described below:

**Table 1:** Participants in the National Oral Health Research Priorities process.

Phase	Objectives	Researchers/ specialists n=46	Decisionmakers n=32	Experts ^a^ n=7	Total
1	Identification of the strategic objectives of MINSA	-	2 ^b^	-	2
2	Identification of research needs regarding oral health	39	-	-	39
3	Expert review and needs assessment according to criteria				
3a	Expert review of needs	-	3 ^b^	3	6
3b	Needs assessment according to criteria	28	14	1	43
4	Prioritization (ranking in the priority list according to rating)	-	3 ^b^	-	3
5	Presentation of priorities	11	29 ^c^	1	41

a Experts appointed by Ministerial Resolution No. 571-2017/MINSA.

b Members of the DSABU/MINSA.

c From to 15 regions.

### Phase 1. Identification of MINSA’s strategic objectives

In the absence of a National Oral Health Plan with strategic objectives, the members of the DSABU identified objectives based on the guidelines of the current MINSA policies, with the methodological guidance of the INS team as shown in Table 2.

**Table 2:** Strategic objectives for the identification and determination of Oral Health Research Priorities 2021-2025.

#	Strategic objectives
1	To develop an epidemiological system of morbidity and associated factors in oral health, for normative support and decision making according to life course.
2	To establish interventions for the prevention, control and treatment of oral pathologies focused on the individual, family and community that impact the quality of life.
3	To strengthen management processes for the implementation of improvement actions in oral health interventions.

### Phase 2. Identification of oral health research needs

This phase was carried out in two stages:

*First Stage. Initial identification*

Thirty-nine of the 46 researchers/specialists (84.8%), identified as key actors, participated in this stage and created the initial statement of research needs; it should be noted that all dental specialties were covered among the 39 participants ^16^, which resulted in proposals from different points of view, complementing each other. As a result of this stage, 220 proposed research needs were obtained (Supplementary Material).

*Second Stage. Classification and grouping*

At this stage, the four members of the SUDENT team carried out all the procedures under consensus, such as the standardization of the wording of the initial proposals, based on five types of research (a. Research to measure the magnitude and distribution of health problems, b. Research to understand the various causes or determinants of the health problem, c. Research to develop solutions or interventions that help prevent and/or mitigate health problems, d. Research to translate the solutions or evidence into practical policies and products, e. Research to evaluate the impact of solutions or interventions) ^9^^,^^17^. Others reclassified the 220 needs according to the strategic objectives and purged the duplicated ones. The wording of these needs was also rephrased, thus obtaining a second list of 32 that were subsequently grouped into 18 proposed needs (Supplementary Material).

### Phase 3. Expert review and needs assessment according to criteria.

Carried out in two stages:

*First Stage. Review of the needs by experts*

The three experts reviewed and suggested adjustments to the wording of some of the proposed needs, and incorporated a couple of needs that had an impact on public health: “i) To develop studies that evaluate the impact of emerging/reemerging diseases related to oral health; and ii) Studies on the prevalence of complications related to receiving or not receiving treatment”. This resulted in the final list of oral health research needs.

*Second stage. Needs assessment according to criteria.*

Once the list of 20 research needs was obtained, it was rated by the participants distributed in the three groups of key actors; but, due to availability issues, it was not possible to have all of them (Table 1). The participant’s rating considered the criteria and assessment scale proposed in the DIIS Methodological Guide (Table 3). An independent descending ordinal list of research needs for each strategic objective was created based on the scores (Table 4).

**Table 3:** Criteria and rating scale for the qualification of National Health Research Priorities ^9^^,^^17^.

Criteria	Rating scale
Knowledge gap: the need to generate knowledge to shorten the distance between the current situation and what it should be; the less studied the subject is, the more it is considered a gap.	1 = very low 2 = low 3 = medium 4 = high 5 = very high
Feasibility: political, technical, financial and operational feasibility (availability of necessary resources, and/or access to them through agreements, alliances or other mechanisms).	
Effects or consequences: impact on the general population and the health system.	

**Table 4:** Sum of Rating of National Oral Health Research Priorities by strategic objective.

#	Strategic objectives	#	Research needs	Rating summation
1	Develop an epidemiological system of morbidity and associated factors in Oral Health, for normative support and decision making according to life course.	1	To develop descriptive epidemiological studies of the conditions and diseases of the stomatognathic system by life course.	535
		2	Studies to identify determinants and factors associated with oral pathologies according to life course.	504
		3	Studies associating systemic and non-communicable diseases with the prevalence and incidence of oral pathologies.	504
		4	Prevalence studies of complications related to receipt or absence of dental treatment.	491
2	Establish interventions for the prevention, control, treatment and maintenance of oral diseases that impact the quality of life, focusing on the individual, family and community.	1	Development of Innovations and/or Information and Communication Technologies (ICT) for oral health interventions (prevention of diseases of the stomatognathic system, promotion, recovery and rehabilitation).	541
		2	Development and evaluation of oral health prevention and promotion programs by life course.	528
		3	Development and evaluation of oral health programs for vulnerable populations.	528
		4	Follow-up and adherence studies to interventions related to oral health.	519
		5	Develop and evaluate the effectiveness of interventions related to oral health.	518
		6	Studies evaluating the impact on systemic health after dental treatment.	518
		7	Studies of methods of dental identification and diagnosis.	483
		8	Experimental *in vitro* studies and/or clinical trials related to oral health.	425
3	Strengthen management processes for the implementation of improvement actions in oral health interventions.	1	Magnitude, distribution and gap of dental human resources.	535
		2	Studies of oral disease impact assessment by life course.	534
		3	Studies of the economic and social impact of access, timeliness and efficacy of dental treatment.	516
		4	Health technology assessment studies in oral health.	510
		5	Economic evaluation studies in oral health.	500
		6	Develop studies that evaluate the impact of emerging/reemerging diseases related to oral health.	498
		7	Develop supply and demand analysis studies for dental services.	491
		8	Studies of quality of care in dental care services in health facilities.	490

### Phase 4. Prioritization (assessment of the priority list according to rating)

The SUDENT technical team and the DSABU participated in this phase, who, by consensus, and after reviewing the needs ratings (Table 4), decided to make modifications that affected objective 2:

Remove need 3 (development and evaluation of oral health programs for vulnerable populations) because it was contained in need 2 of said objective (development and evaluation of oral health prevention and promotion programs by life stage).

Regroup research need 5 (develop and evaluate the efficacy of interventions related to oral health) and need 8 (*in vitro* experimental studies and/or clinical trials related to oral health), which was ranked last (Table 4); however, they were regrouped given their relevance and methodological level, so that the final wording was “develop experimental studies and/or evaluation of efficacy of interventions related to oral health”.

After the proposed modifications, the first four research needs were selected for each objective, thus obtaining the final list of 12 research priorities (Table 5).

**Table 5:** National Oral Health Research Priorities 2022-2026 approved by Ministerial Resolution No. 262-2022/MINSA.

Strategic Objective	Research needs
Develop an epidemiological system of morbidity and associated factors in oral health, for normative support and decision making according to life course.	To develop descriptive epidemiological studies of the conditions and diseases of the stomatognathic system by life course.
	Studies to identify determinants and factors associated with oral pathologies according to life course.
	Studies associating systemic and non-communicable diseases with the prevalence and incidence of oral pathologies.
	Prevalence studies of complications related to receiving or not receiving dental treatment.
Establish interventions for the prevention, control, treatment and maintenance of oral diseases that impact the quality of life, focusing on the individual, family and community.	Development of Innovations and/or Information and Communication Technologies (ICT) for oral health interventions (prevention of diseases of the stomatognathic system, health promotion, recovery and rehabilitation).
	Development and evaluation of oral health prevention and promotion programs by life course.
	Follow-up and adherence studies to interventions related to oral health.
	To develop experimental and/or efficacy evaluation studies of interventions related to oral health.
Strengthen management processes for the implementation of improvement actions in oral health interventions.	Magnitude, distribution and gap of dental human resources.
	Life course oral disease impact assessment studies.
	Studies of oral disease impact assessment by life course.
	Health Technology Assessment Studies in oral health.

### Phase 5. Presentation of priorities

SUDENT presented the final list of the National Oral Health Research Priorities to the key actors of the process (researchers/specialists, experts and decision makers); then, the bilateral administrative procedures were carried out between the INS and MINSA for its approval, and subsequently it was made official through Ministerial Resolution No. 262-2022/MINSA dated March 30, 2022, and considered valid for the 2022-2026 period ^18^, with a horizon of five years as in previous processes ^10^^,^^19^^,^^20^. Due to time availability, not all actors were able to participate in the presentation event held on June 25, 2021; however, representatives of the regions of Junin, Ica and Loreto attended virtually, in addition to the coordinator of the oral health strategy, with 29 participants from 15 regions (Table 1).

## RESULTS AND COMPARISONS

Research priorities are defined by the INS as “research needs prioritized on the basis of the health problems that most affect the population and that require effective and efficient responses” ^21^. This important work has been carried out by the INS in several opportunities, which materialized in Ministerial Resolutions such as Ministerial Resolution No. 262-2022/MINSA ^18^ that approves the “National Research Priorities in Oral Health” (Table 5), which are the subject of this article.

The National Institute of Dental and Craniofacial Research of the United States, known as NIDCR, developed its 2021-2026 Strategic Plan based on four guiding principles and five strategic priorities with their respective objectives ^22^. Similarly, the National Institute for Health Research (NIHR) in England has established 10 oral health priorities ^23^. When reviewing the plan and the questions identified by the English community, some similarities were found between their objectives and the priorities identified in Peru, ^18^; the main difference between both processes and ours being the participants. This process was carried out with researchers/specialists, decision-makers and experts (there was no participation of the civil society); while in the USA, several entities related to the scientific community, federal agencies and industry participated; and in England, the NIHR group participated in collaboration with the dental schools and public health in England to identify unanswered questions related to oral health research for patients, the public, caregivers and oral health professionals.

On the other hand, the 2018 priority identification study in Iran, a developing country like ours, reported 171 topics in six areas related to Oral Health, obtaining 44 topics on “dental clinical care and treatment”, 37 topics on “health and prevention”, 19 topics on “health promotion and education”, 37 topics on “management of dental care delivery”, 16 topics on “policies and their requirements” and 18 topics on “dental materials, equipment and technologies” ^24^, topics that largely coincide with those identified in Peru ^18^.

Among Latin American countries, Brazil considered oral health in its National Health Research Priorities Agenda in 2015, whose subagenda coincides with the National Oral Health Research Priorities of Peru in the topics related to epidemiology, risk factors, development of the epidemiological system, development of strategies for oral health promotion, innovations for treatment and supply of oral health services ^25^.

The Oral Health Research Priorities identified in Peru are in line with the research priorities for strengthening the Global Health Action for Oral Health, which are rooted in the knowledge gaps in: i) Epidemiology and health information systems for the surveillance of oral conditions; ii) Collection, harmonization and rigorous evaluation of evidence for equity in the prevention and treatment of oral conditions; and iii) Strategies to provide essential quality oral health care without financial barriers ^26^.

## EXPECTED IMPACT

The oral health situation in Peru is a public health problem, as it ranks second among the 10 leading causes of morbidity in the three natural regions (coast, highlands and jungle) of the country ^27^. The health emergency caused by the COVID-19 pandemic has aggravated the oral health of the Peruvian population. The restrictions on access to health services, especially dental care, adopted to mitigate contagion ^28^^-^^31^ showed the need for the identification and implementation of new health interventions, guidelines and public policies in the field of oral health, taking into account the current context. Therefore, the “National Oral Health Research Priorities 2022-2026” ^18^ have a transcendental role in guiding future research on oral health and will in turn serve to redefine policies and establish and/or reformulate more effective programs ^32^ that will contribute to the improvement of the health system ^33^, in accordance with Law No. 31540, which declares the formulation and implementation of the national oral health policy to be of national interest and public necessity ^34^.

## LESSONS LEARNED, PERSPECTIVES AND RECOMMENDATIONS

It should also be noted that during this process, decision-makers in the public management of oral health at both central and regional levels, who may change according to the political context, were considered as actors; therefore, it was important to have the vision of the academia, represented by professionals who evidence the conduct of research.

It is relevant to understand the social context in which the process was carried out, since there were some restrictions for face-to-face coordination due to the COVID-19 pandemic. All coordination and activities were carried out virtually, which did not guarantee full participation of the initially considered actors; however, all actors in all stages and phases of the process participated, which allowed having different points of view.

Regarding the conduct of the process by the SUDENT team, it should be noted that all actions involving changes in the proposals were carried out under consensus and subsequently reviewed in the subsequent stage for acceptance.

The process complied with the guidelines established in the Methodological Guide of the DIIS ^9^ under the technical advice of the DIIS (which has carried out previous processes of identification of national health research priorities). Therefore, pre-publication of the list of priorities was not possible because throughout the process, criticism and recommendations were received from the invited key actors, whose contributions and comments were openly valued. However, the omission of pre-publication, as well as the declaration of conflicts of interest, could be considered as limitations.

Another limitation in this process was related to the difficulties of the key actors to continually participate in all phases and activities, which was related to aspects of time availability; however, it should be noted that this difficulty was mitigated to a greater extent by conducting the process virtually. Regarding the varying participation rate, we must recognize potential participation bias due to voluntariness, which could represent a potential motivation that was not declared as a conflict of interest and could not be identified or noticed.

Selection bias is also a possibility, given that although the list of researchers with RENACYT code (30 researchers according to the download date) was used to contact the key actors of the group that represents the researchers, and although most of them provided their email address, not all of them could be reached. Therefore, they were contacted through their contact network, social networks and/or WhatsApp; however, some did not respond, the line was out of service, or they were not available. In view of this situation, dental surgeons who work as teachers and direct the development of research protocols at universities were included. Specialists, who apply the results of the research directly on the patient were also included. Therefore, the representativeness of the oral health scientific community may have been affected.

In conclusion, this article has described the process followed for the identification, for the first time, of the Oral Health Research Priorities in Peru, which are valid from 2022 to 2026 and will guide the development of research that will be used as evidence for decision-making in oral health, in the hope that this process will serve as a model for the identification of a new list of research priorities in this field, being perfected each time.

After identifying priorities on the basis on the National Multisectoral Health Policy 2030 ^35^, the published report on Global Oral Health by the World Health Organization ratified that oral health requires urgent action and highlights the challenges and opportunities for oral health ^36^ that should be considered for research aligned with the priorities identified during this process.

We call on all oral health research professionals to contribute to the development of Peruvian scientific production framed within these priorities, thus contributing with scientific evidence that will support solid public policies for the benefit of Peruvian society. In addition, we recommend the DIIS to evaluate the relevance of incorporating the pre-publication of results and the declaration of conflicts of interest as part of the methodology for future processes.

Similarly, it is necessary to promote the implementation of the National Priorities for Oral Health Research in the academia, in order to encourage its development in universities throughout the country; this will also allow the competent bodies to include the topic of oral health in the competitive funds encouraging researchers to develop research that require a larger budget, such as clinical trials and technological innovations, considering the importance of funding for research of the highest quality, taking the National Institutes of Health as a model ^37^. The priorities identified in terms of their approach represent a macro level for the planning and management of oral health research, which should help public and private institutions, as well as the academia, in order to formulate research topics/lines/ideas, which in a more specific way will promote the formulation of research questions that are aligned with the needs to generate evidence and guarantee an adequate use of the limited resources available.
