# Supplementary material for: National priorities for oral health research, Peru 2022-2026: process, experiences and perspectives
Source: Rev Peru Med Exp Salud Publica. 2023 Sep 28;40(3):354–63. doi: 10.17843/rpmesp.2023.403.12082 (PMC10953641; doi:10.17843/rpmesp.2023.403.12082)
Supplement: Supplementary material. — Available in the electronic version of the RPMESP. [file rpmesp-40-03-12082-s001.docx]

**Objetivo 1.**

| **#** | **Listado original** | **V°B°** | **Clasificación o Reformulación** | **Objetivo** |
| --- | --- | --- | --- | --- |
| **1** | **Determinar la prevalencia de caries dental** | **SI** | **Desarrollar estudios epidemiológicos en salud bucal** | **1** |
| **2** | **Necesidad de un Mapeo Nacional que evalúe la concentración natural de fluoruros en el agua a nivel nacional** | **SI** | **Estudios que evalúen la eficacia de las intervenciones en salud bucal** | **2** |
| **3** | **Características epidemiológicas de los pacientes con infecciones odontogénicas** | **SI** | **Desarrollar estudios epidemiológicos en salud bucal** | **1** |
| **4** | **Prevalencia de las lesiones de caries no tratadas en infantes, niños y adolescentes** | **SI** | **Desarrollar estudios epidemiológicos en salud bucal** | **1** |
| **5** | **Estudios nacionales sobre la introducción del azúcar en la dieta del infante y consumo de azúcar en las familias peruanas.** | **SI** | **Estudios que evalúen la eficacia de las intervenciones en salud bucal** | **1** |
| **6** | **Levantar el mapa epidemiológico de Salud bucal e identificar las poblaciones más afectadas.** | **SI** | **Desarrollar estudios epidemiológicos en salud bucal** | **1** |
| **7** | **CARIES DENTAL POR GRUPO DE EDADES DE 0-5, 6-12 Y 13-18** | **SI** | **Desarrollar estudios epidemiológicos en salud bucal** | **1** |
| **8** | **Alteraciones orofaciales en bebes prematuros, prevalencia de caries en infantes, niños, adolescentes con discapacidad** | **SI** | **Desarrollar estudios epidemiológicos en salud bucal** | **1** |
| **9** | **Estudio epidemiológico de las infecciones odontogénicas** | **SI** | **Desarrollar estudios epidemiológicos en salud bucal** | **1** |
| **10** | **Estudios epidemiológicos de las principales enfermedades bucales en las diferentes regiones del Perú** | **SI** | **Desarrollar estudios epidemiológicos en salud bucal** | **1** |
| **11** | **Epidemiología de las enfermedades periodontales y su relación con enfermedades sistémicas** | **SI** | **Desarrollar estudios epidemiológicos en salud bucal** | **1** |
| **12** | **Estudios epidemiológicos de enfermedades bucales de alta prevalencia por etapas de vida.** | **SI** | **Desarrollar estudios epidemiológicos en salud bucal** | **1** |
| **13** | **Estudio epidemiológico de las Disfunción Temporomandibular** | **SI** | **Desarrollar estudios epidemiológicos en salud bucal** | **1** |
| **14** | **Prevalencia e incidencia de enfermedad periodontal y factores de riesgo, según etapas de vida.** | **SI** | **Estudios para identificar factores asociados en patología bucales según curso de vida** | **1** |
| **15** | **Epidemiología de caries dental en el Perú con métodos de alta sensibilidad / especificidad (ICDAS/CAST), con examinadores calibrados en una muestra nacional** | **SI** | **Desarrollar estudios epidemiológicos en salud bucal** | **1** |
| **16** | **construcción y mantenimiento de mapas epidemiológicos nacionales sobre enfermedades en salud bucal** | **SI** | **Desarrollar estudios epidemiológicos en salud bucal** | **1** |
| **17** | **Mapeo epidemiológico de enfermedades más prevalentes.** | **SI** | **Desarrollar estudios epidemiológicos en salud bucal** | **1** |
| **18** | **Realización de censos epidemiológicos de enfermedades periodontales y periimplantarias** | **SI** | **Desarrollar estudios epidemiológicos en salud bucal** | **1** |
| **19** | **Estudios epidemiológicos de enfermedades bucales de baja prevalencia por etapas de vida.** | **SI** | **Desarrollar estudios epidemiológicos en salud bucal** | **1** |
| **20** | **Evaluación de la prevalencia de patologías del sistema estomatognático (caries dental, enfermedad periodontal, DTM y neoplasias) etapas de vida y/o condiciones sistémicas** | **SI** | **Desarrollar estudios epidemiológicos en salud bucal** | **1** |
| **21** | **Prevalencia de las alteraciones en el desarrollo maxilofacial** | **SI** | **Desarrollar estudios epidemiológicos en salud bucal** | **1** |
| **22** | **Epidemiología de enfermedades periodontales y periimplantarias** | **SI** | **Desarrollar estudios epidemiológicos en salud bucal** | **1** |
| **23** | **Sistema epidemiológico de morbilidad de las patologías pulpares y periapicales** | **SI** | **Desarrollar estudios epidemiológicos en salud bucal** | **1** |
| **24** | **Realizar un estudio epidemiológico a nivel Nacional de las enfermedades periodontales en la población peruana.** | **SI** | **Desarrollar estudios epidemiológicos en salud bucal** | **1** |
| **25** | **evaluación de la salud bucal nacional cada 10 años** | **SI** | **Desarrollar estudios epidemiológicos en salud bucal** | **1** |
| **26** | **Estudio epidemiológico de caries dental / dolor /pérdida dentaria asociado a calidad de vida relacionado a salud bucal** | **SI** | **Desarrollar estudios epidemiológicos en salud bucal** | **1** |
| **27** | **Creación de una base de datos sobre las patologías del del sistema estomatognático según regiones** | **SI** | **Desarrollar estudios epidemiológicos en salud bucal** | **1** |
| **28** | **Hacer los autoreportes epidemiológicos** | **SI** | **Desarrollar estudios epidemiológicos en salud bucal** | **1** |
| **29** | **Prevalencia de anomalías dentoesqueléticas en la población con Apnea Obstructiva del Sueño** | **SI** | **Desarrollar estudios epidemiológicos en salud bucal** | **1** |
| **30** | **Epidemiología de la caries dental** | **SI** | **Desarrollar estudios epidemiológicos en salud bucal** | **1** |
| **31** | **Epidemiología de las maloclusiones** | **SI** | **Desarrollar estudios epidemiológicos en salud bucal** | **1** |
| **32** | **Obtener información base de datos** | **SI** | **Desarrollar estudios epidemiológicos en salud bucal** | **1** |
| **33** | **Ubicar las regiones con mayor tasa de prevalencia de caries dental** | **SI** | **Desarrollar estudios epidemiológicos en salud bucal** | **1** |
| **34** | **Factores de riesgo para caries dental en infantes, niños y adolescentes en el Perú** | **SI** | **Estudios para identificar factores asociados en patología bucales según curso de vida** | **1** |
| **35** | **Determinar factores de riesgo de caries dental en población infantil con discapacidad** | **SI** | **Estudios para identificar factores asociados en patología bucales según curso de vida** | **1** |
| **36** | **Monitorear periódicamente la calidad y distribución de la sal fluorada en el territorio nacional enfatizando su alcance a comunidades vulnerables y sin presencia de concentraciones de concentraciones de fluoruro en el agua con alcance preventivo/terapéutico** | **SI** | **Estudios que evalúen la eficacia de las intervenciones en salud bucal** | **3** |
| **37** | **Determinar que variables y factores en la toma de datos nacionales** | **NO** | **Impreciso** |  |
| **38** | **FACTORES ASOCIADOS INFECCIONES ODONTOGÉNICAS** | **SI** | **Estudios para identificar factores asociados en patología bucales según curso de vida** | **1** |
| **39** | **FACTORES DE RIESGO ASOCIADOS A CARIES DENTAL** | **SI** | **Estudios para identificar factores asociados en patología bucales según curso de vida** | **1** |
| **40** | **Estudios de cohorte con muestras poblacionales que puedan evaluar factores de riesgo relacionados a las enfermedades bucales más frecuentes. Estudios de cohorte de nacimiento** | **SI** | **Estudios para identificar factores asociados en patología bucales según curso de vida** | **1** |
| **41** | **Relación causa efecto de las infecciones odontogénicas en la prevalencia de enfermedades cardiovasculares y metabólicas en la población peruana.** | **SI** | **Estudios que asocien enfermedades sistémicas y la salud bucal** | **1** |
| **42** | **Factores de riesgo para el desarrollo de infecciones odontogénicas en la población peruana.** | **SI** | **Estudios para identificar factores asociados en patología bucales según curso de vida** | **1** |
| **43** | **Evaluar no sólo según etapas de vida si no también según distintas realidades** | **NO** | **Impreciso** |  |
| **44** | **Establecer e identificar factores de riesgo locales asociados al ambiente como clima, altura, nutrición, nivel educacional, entre otros, para determinar las poblaciones con mayor riesgo de padecer periodontitis y en consecuencia otras enfermedades crónicas no transmisibles.** | **SI** | **Estudios para identificar factores asociados en patología bucales según curso de vida** | **1** |
| **45** | **Enfermedad periodontal como factor de riesgo de enfermedades sistémicas consideradas de prioridad de investigación en salud: enfermedades metabólicas y cardiovasculares, enfermedades respiratorias (COVID-19, asma, neumonía, EPOC), malnutrición, etc.** | **SI** | **Estudios que asocien enfermedades sistémicas y la salud bucal** | **1** |
| **46** | **Relación entre las enfermedades periodontales y periimplantarias con enfermedades y/o condiciones sistémicas** | **SI** | **Estudios que asocien enfermedades sistémicas y la salud bucal** | **1** |
| **47** | **Determinar los principales factores de riesgo en cada región** | **SI** | **Estudios para identificar factores asociados en patología bucales según curso de vida** | **1** |
| **48** | **Determinar los niveles de flúor que existe en el agua de las diferentes regiones del país, lo que permitirá establecer un programa de reforzamiento y control más sostenible.** | **SI** | **Estudios para identificar factores asociados en patología bucales según curso de vida** | **1** |
| **49** | **Reconocer las consecuencias de pérdida de dientes a nivel de la población** | **SI** | **Desarrollo y evaluación de programas de promoción** | **2** |
| **50** | **Calidad de vida - problemas bucales emergentes** | **SI** | **Estudios que evalúen enfermedades emergentes relacionados a la salud bucal** | **3** |
| **51** | **determinar prevalencia de alteraciones en la estructura del esmalte asi como los factores etiológicos que las producen** | **SI** | **Desarrollar estudios epidemiológicos en salud bucal** | **1** |
| **52** | **Estudios sobre epidemiología, morbilidad y factores de la infección odontogénica según etapas de vida, morbilidades asociadas y severidad** | **SI** | **Desarrollar estudios epidemiológicos en salud bucal** | **1** |
| **53** | **Prevalencia de la obesidad cervical y anomalías dentoesqueléticas como factores predisponentes a patología del sueño** | **SI** | **Desarrollar estudios epidemiológicos en salud bucal** | **1** |
| **54** | **Estudio epidemiológico de prevalencia de periodontitis incluyendo la presencia de factores de riesgo por otros especialistas, con el fin de determinar de forma escalonada el riesgo para cada tipo de paciente.** | **SI** | **Desarrollar estudios epidemiológicos en salud bucal** | **1** |
| **55** | **Estudio epidemiológico de las enfermedades más prevalentes y sus factores de riesgo** | **SI** | **Desarrollar estudios epidemiológicos en salud bucal** | **1** |
| **56** | **Epidemiologia de la gingivitis y periodontitis con metodologías recomendadas por los epidemiólogos para evitar subestimar. hacerla identificando los factores asociados como educación, tabaquismo, enfermedades sistémicas, hábitos, etc.** | **SI** | **Desarrollar estudios epidemiológicos en salud bucal** | **1** |
| **57** | **Establecer los principales factores de prevalencia de la caries dental** | **SI** | **Desarrollar estudios epidemiológicos en salud bucal** | **1** |
| **58** | **Establecer estudios de prevalencia de las maloclusiones dentarias en las diferentes etapas de la vida que puedan alterar el correcto funcionamiento del sistema estomatognático** | **SI** | **Desarrollar estudios epidemiológicos en salud bucal** | **1** |
| **59** | **Realizar estudios observacionales, en un primer momento, en pacientes con enfermedad periodontal y que enfermedades sistémicas padece.** | **SI** | **Desarrollar estudios epidemiológicos en salud bucal** | **1** |
| **60** | **Diagnóstico prioritario de las enfermedades periodontales** | **NO** | **Capacitación** |  |
| **61** | **Composición del microbioma de las infecciones de origen odontogénicos** | **SI** | **Estudios experimentales in vitro** | **1** |
| **62** | **Variantes morfológicas de los maxilares y mandíbula con fines de identificación forense, la pandemia nos ha demostrado que es importante contar con registros más precisos para la identificación de personas.** | **SI** | **Estudio de métodos de identificación y/o diagnóstico odontológico** | **2** |
| **63** | **Estudio de las teorías y métodos para la prevención de enfermedades periodontales** | **SI** | **Estudios para identificar factores asociados en patología bucales según curso de vida** | **1** |
| **64** | **Brechas de especialistas en endodoncia en los 3 niveles de atención de salud para su progresivo cierre** | **SI** | **Magnitud, distribución y brecha de recursos humanos odontológicos** | **3** |
| **65** | **Individualización de estrategias de intervención para prevención por región y por grupo etario** | **SI** | **Desarrollo y evaluación de programas de promoción** | **2** |
| **66** | **Grado de conocimiento sobre las competencias del cirujano dentista de práctica general y los especialistas ejem: competencias del cirujano bucal y maxilofacial** | **NO** | **Normativa** |  |
| **67** | **Rol de la saliva y Sars-Cov2** | **SI** | **Estudios experimentales in vitro** | **1** |
| **68** | **cuáles son los factores asociados** | **NO** | **Impreciso** |  |

**Objetivo 2.**

| **#** | **Necesidades de investigación** | **V° B°** | **Clasificación o Reformulación** | **Objetivo** |
| --- | --- | --- | --- | --- |
| **1** | **Diagnóstico precoz del cáncer oral** | **NO** | **Capacitación** |  |
| **2** | **Guía clínica para la prescripción de exámenes radiográficos en Odontología tanto para los exámenes radiográficos convencionales bidimensionales como para Tomografía Computarizada Volumétrica.** | **NO** | **Guías de Práctica Clínica en Rx** |  |
| **3** | **Capacitar al personal de atención primaria en las técnicas de mínima intervención en caries dental** | **NO** | **Capacitación** |  |
| **4** | **Establecer los factores de riesgo para enfermedades bucales por etapas de vida** | **SI** | **Factores de riesgo** | **1** |
| **5** | **Programas de SB en escuelas e instituciones** | **SI** | **Prevención, promoción y Tratamiento** | **2** |
| **6** | **diseño de guías para niños con enfermedades sistémicas** | **NO** | **Guías de Práctica Clínica** |  |
| **7** | **Priorizar las Intervenciones con otros profesionales de salud a nivel materno-infantil** | **NO** | **Políticas** |  |
| **8** | **Tratamiento quirúrgico y farmacológico de las infecciones odontogénicas** | **SI** | **Prevención, promoción y Tratamiento** | **2** |
| **9** | **Innovación en materiales para prevención, control y tratamiento desde el diagnostico epidemiológico de enfermedades de salud bucal** | **SI** | **Desarrollar estudios epidemiológicos en salud bucal** | **1,2** |
| **10** | **EVALUACIÓN DEL IMPACTO DAS MEDIDAS DE USO COMUNITARIO** | **SI** | **Evaluación de impacto** | **3** |
| **11** | **Eficacia de los programas nutricionales escolares nacionales usados en programas sociales en relación con la salud bucal de los escolares** | **SI** | **Prevención, promoción y Tratamiento** | **2** |
| **12** | **Entrevista motivacional como estrategia de prevención de caries de infancia temprana (Visita al dentista antes del primero año, dieta e higiene oral)** | **SI** | **Prevención, promoción y Tratamiento** | **2** |
| **13** | **Capacitar al personal primario de salud en salud bucal y a los cirujanos dentistas en la atención de infantes** | **NO** | **Capacitación** |  |
| **14** | **Programas de SB en escuelas e instituciones** | **SI** | **Prevención, promoción y Tratamiento** | **2** |
| **15** | **TRATAMIENTO ADECUADO PARA INFECCIONES ODONTOGÉNICAS** | **SI** | **Prevención, promoción y Tratamiento** | **2** |
| **16** | **Tratamiento Restaurador atraumático como estrategia: preventiva, interceptiva y terapéutica en instituciones de salud y programas extramurales.** | **SI** | **Prevención, promoción y Tratamiento** | **2** |
| **17** | **SDF -(TRA )** | **SI** | **Prevención, promoción y Tratamiento** | **2** |
| **18** | **Técnicas de mínima intervención -Capacitación y evaluación** | **SI** | **Estudios que evalúen la eficacia de las intervenciones en salud bucal** | **2** |
| **19** | **Determinar prioridades en las campañas de intervención por regiones** | **NO** | **Políticas** |  |
| **20** | **Necesidad de estudios longitudinales que evalúen la efectividad de programas de salud bucal y su impacto en la calidad de vida** | **SI** | **Estudios que evalúen la eficacia de las intervenciones en salud bucal** | **2** |
| **21** | **Impacto del COVID en la salud Bucal** | **SI** | **Impacto de enfermedades infecciosas relacionadas a la cavidad bucal** | **3** |
| **22** | **Periodoncia e interdisciplinariedad con otras enfermedades crónicas no trasmisibles** | **SI** | **Estudios que asocien enfermedades sistémicas y la salud bucal** | **1** |
| **23** | **Fluorizar como medio preventivo con agentes fluorados seleccionados basándose en la evidencia** | **SI** | **Prevención, promoción y Tratamiento** | **2** |
| **24** | **Prevalencia de patologías de ATM y trastornos del sueño en pacientes con deformidades dentofaciales clase II no tratados.** | **SI** | **Desarrollar estudios epidemiológicos en salud bucal** | **1** |
| **25** | **Prevalencia de patologías de ATM y trastornos del sueño en pacientes con deformidades dentofaciales clase II tratamiento solo con ortodoncia.** | **SI** | **Desarrollar estudios epidemiológicos en salud bucal** | **1** |
| **26** | **Estudio sobre la prevención control y tratamiento de las infecciones odontogénicas** | **SI** | **Prevención, promoción y Tratamiento** | **2** |
| **27** | **Estudio sobre la prevención, control y tratamiento de la disfunción Temporomandibular** | **SI** | **Prevención, promoción y Tratamiento** | **2** |
| **28** | **Prevalencia y factores asociados de la xerostomía** | **SI** | **Factores de riesgo** | **1** |
| **29** | **Establecer programas de educación en salud bucal, enfatizando en las técnicas de higiene bucal** | **SI** | **Prevención, promoción y Tratamiento** | **2** |
| **30** | **Implementación de programas educativos que promuevan la salud periodontal y periimplantaria** | **SI** | **Prevención, promoción y Tratamiento** | **2** |
| **31** | **Control de caries y enfermedad periodontal desde los primeros meses de vida, alianzas estratégicas con las diferentes especialidades de salud, como la enfermería, medicina, obstetricia, nutrición, gerontología.** | **NO** | **Políticas** |  |
| **32** | **Tratamiento farmacológico del dolor e inflamación postquirúrgicos** | **SI** | **Prevención, promoción y Tratamiento** | **2** |
| **33** | **Desde el diagnostico etario, regional y psicosocial establecimiento de estrategias de intervención para prevención, control tratamiento** | **SI** | **Prevención, promoción y Tratamiento** | **2** |
| **34** | **Prevención, control y tratamiento interdisciplinario de las enfermedades periodontales y periimplantarias** | **SI** | **Prevención, promoción y Tratamiento** | **2** |
| **35** | **Intervenciones en prevención y tratamiento. educación y la intervención en los niveles de atención primaria** | **SI** | **Prevención, promoción y Tratamiento** | **2** |
| **36** | **Estrategias para el manejo de dolor en pacientes con lesiones pulpares y periapicales principalmente en pulpitis irreversible sintomática, periodontitis apical sintomática y absceso apical agudo localizado** | **SI** | **Prevención, promoción y Tratamiento** | **2** |
| **37** | **Evaluación de la política nacional de sal fluorada.** | **SI** | **Estudios que evalúen la eficacia de las intervenciones en salud bucal** | **2,3** |
| **38** | **Estudios clínicos/de intervención que evalúen la eficacia de productos utilizados en prevención/control de caries dental** | **SI** | **Estudios que evalúen la eficacia de las intervenciones en salud bucal** | **2** |
| **39** | **Rol de los colutorios (cpc) en la reducción de la carga viral del Sars-Cov-2** | **SI** | **Prevención, promoción y Tratamiento** | **2** |
| **40** | **Prevención, control y tratamiento de las enfermedades periodontales y periimplantarias** | **SI** | **Prevención, promoción y Tratamiento** | **2** |
| **41** | **Diseño de un programa de salud bucal orientado a la promoción y prevención de patologías pulpares y periapicales en los hogares de mayor riesgo** | **SI** | **Prevención, promoción y Tratamiento** | **2** |
| **42** | **Estudio sobre la GPC, prevención control y tratamiento de las infecciones odontogénicas,** | **SI** | **Prevención, promoción y Tratamiento** | **2** |
| **43** | **Enfoque en la prevención y tratamiento de las maloclusiones dentarias** | **SI** | **Prevención, promoción y Tratamiento** | **2** |
| **44** | **Intervenciones a nivel de médicos generales en hacer descarte de periodontitis.** | **SI** | **Prevención, promoción y Tratamiento** | **2** |
| **45** | **Realizar un programa de cumplimiento de la fase de mantenimiento en pacientes con enfermedad periodontal ya tratada.** | **SI** | **Seguimiento y adherencia** | **2** |
| **46** | **Estudio de políticas nacionales sobre alimentación saludable, efecto en las enfermedades no transmisibles de los programas de alimentación** | **SI** | **Estudios que evalúen la eficacia de las intervenciones en salud bucal** | **2** |
| **47** | **Evaluación de la implementación de las guías de prácticas clínicas vigentes.** | **NO** | **Capacitación** |  |
| **48** | **Uso de pasta dental fluorada por grupos de edades** | **SI** | **Prevención, promoción y Tratamiento** | **2** |
| **49** | **Determinar el impacto del tratamiento periodontal frente a los problemas de salud considerados como prioridades de investigación nacional.** | **SI** | **Evaluación de impacto** | **3** |
| **50** | **Fomento de educación de la salud bucal a todas las familias del Perú de forma mensual utilizando los medios de comunicación como la televisión, radio y zoom.** | **SI** | **Prevención, promoción y Tratamiento** | **2** |
| **51** | **Estudios que determinen la eficacia de las soluciones irrigantes para la limpieza de los conductos radiculares (concentración de las soluciones)** | **SI** | **Estudios que evalúen la eficacia de las intervenciones en salud bucal** | **2** |
| **52** | **Valorar la eficacia de intervenciones para el tratamiento de gingivitis, periodontitis y enfermedades periimplantarias** | **SI** | **Estudios que evalúen la eficacia de las intervenciones en salud bucal** | **2** |
| **53** | **Formulación de productos para la prevención de caries dental** | **SI** | **Prevención, promoción y Tratamiento** | **2** |
| **54** | **Prevención y tratamiento de la caries dental** | **SI** | **Prevención, promoción y Tratamiento** | **2** |
| **55** | **Calibrar y capacitar a los odontólogos generales en el diagnóstico de la periodontitis y la necesidad de tratamiento periodontal, para su oportuna derivación a especialistas.** | **NO** | **Capacitación** |  |
| **56** | **Realizar estudios sobre tipos de medicamentos utilizados por la población y el grado de enfermedad periodontal que padece.** | **SI** | **Prevención, promoción y Tratamiento** | **2** |
| **57** | **Promover el uso del odontograma como un medio de identificación de las personas, el cual debe realizarse cada 6 meses y debe estar incluido en los datos biométricos de cada peruano. Sustento Ley general de salud, ley del trabajo del cirujano dentista.** | **SI** | **Método de identificación y Diagnóstico odontológico** | **2** |
| **58** | **Tratamiento de caries de mínima intervención: remoción selectiva de tejido cariado** | **SI** | **Prevención, promoción y Tratamiento** | **2** |
| **59** | **Estudios longitudinales para la prevención y tratamiento** | **SI** | **Prevención, promoción y Tratamiento** | **2** |
| **60** | **Diseño de un programa de salud bucal orientado a la promoción y prevención de patologías pulpares y periapicales en centros de formación colegios, universidades entre otros** | **SI** | **Prevención, promoción y Tratamiento** | **2** |
| **61** | **Tratamiento inmunológico de las enfermedades periodontales resistentes / Mantenimiento periodontal y su importancia en la salud general** | **SI** | **Prevención, promoción y Tratamiento** | **2** |
| **62** | **Identificar a la población de niños y adolescentes con sobrepeso y deformidades dentofaciales.** | **SI** | **Desarrollar estudios epidemiológicos en salud bucal** | **1** |
| **63** | **Formulación de productos accesibles a la población para el control de la pérdida de tejido dentario** | **SI** | **Prevención, promoción y Tratamiento** | **2** |
| **64** | **Estudio sobre la legislación actual y mejoras en las tazas de impuestos a alimentos azucarados y su distribución en la población infantil** | **NO** | **Políticas normativas** |  |
| **65** | **Nivel de conocimiento de los profesionales para manejo de enfermedades odontogénicas** | **NO** | **Capacitación** |  |
| **66** | **Prevención y tratamiento de las maloclusiones** | **SI** | **Prevención, promoción y Tratamiento** | **2** |
| **67** | **Implementación de pruebas de tamizaje en encuestas nacionales** | **SI** | **Método de identificación y Diagnóstico odontológico** | **2** |
| **68** | **Determinar la historia natural de la enfermedad periodontal, los niveles de prevención y actividades de salud pública específicamente dirigidas a cada nivel.** | **SI** | **Prevención, promoción y Tratamiento** | **2** |
| **69** | **Capacitar en varias sesiones a odontólogos generales en raspado y alisado radicular y desinfección de boca completa con el fin que puedan disminuir efectivamente la carga microbiana y niveles inflamatorios de los pacientes afectados por periodontitis y otras enfermedades crónicas no transmisibles.** | **NO** | **Capacitación** |  |
| **70** | **Elaboración de GPC para el diagnóstico, tratamiento y mantenimiento de patologías del sistema estomatognático según condiciones sistémicas** | **NO** | **Práctica Clínica** |  |
| **71** | **Prevalencia de patologías ginecológicas y disfunción temporomandibular.** | **SI** | **Desarrollar estudios epidemiológicos en salud bucal** | **1** |
| **72** | **Evaluar las asociaciones gubernamentales y no gubernamentales para la prevención de Caries dental** | **SI** | **Prevención, promoción y Tratamiento** | **2** |
| **73** | **Repercusión de políticas nacionales en salud bucal en la población** | **SI** | **Evaluación de impacto** | **3** |
| **74** | **Conocer las patologías bucales más frecuentes** | **SI** | **Desarrollar estudios epidemiológicos en salud bucal** | **1** |
| **75** | **Diseño de programa de salud bucal para personas con discapacidad** | **SI** | **Prevención, promoción y Tratamiento** | **2** |
| **76** | **Impacto del estado de salud bucal relacionado a la calidad de vida en las personas con discapacidad por curso de vida.** | **SI** | **Evaluación de impacto** | **3** |

**Objetivo 3.**

| **#** | **Listado original** | **V°B°** | **Clasificación o Reformulación** | **Objetivo** |
| --- | --- | --- | --- | --- |
| **1** | **Estudios que evalúen el uso de prototipos tecnológicos para impartir conocimientos relacionados a cuidados, prevención de enfermedades bucales.** | **SI** | **Desarrollo de TICS para actividades de prevención, promoción y control de enfermedades de salud bucal** | **2** |
| **2** | **Comenzar a realizar estudios relacionados a costo-eficacia de tratamientos o programas de salud bucal** | **SI** | **Estudios de costo-efectividad de las intervenciones en salud bucal** | **3** |
| **3** | **Trabajar con grupos con más prioridad nacional (materno infantil) generando equipos multidisciplinarios** | **NO** | **Políticas** |  |
| **4** | **Incluir unidad de campo (postas, etc.) a nivel de serumista/doctor incentivando investigación y salud publica en jóvenes profesionales** | **NO** | **Políticas** |  |
| **5** | **Conocer el impacto de la educación para la salud bucal en infancia niñez adolescencia y padres de familia** | **SI** | **Estudios que evalúen la eficacia de las intervenciones en salud bucal** | **3** |
| **6** | **Conocer el impacto de la educación para la salud bucal usando Tics en infancia niñez adolescencia y padres de familia** | **SI** | **Estudios que evalúen la eficacia de las intervenciones en salud bucal** | **3** |
| **7** | **Desarrollo del aplicativo móvil (Proyecto Sonia Apaza) para incrementar el "alfabetismo de salud bucal" para incluir al paciente en el cuidado de su propia salud.** | **SI** | **Desarrollo de TICS para actividades de prevención, promoción y control de enfermedades de salud bucal** | **2** |
| **8** | **Flujo de atención con la cartilla de salud bucal "Programa Salvadientes" para la inclusión de la salud bucal en las estrategias de salud general del infante conducido por las enfermeras de los centros de salud periféricos.** | **SI** | **Estudios que evalúen la eficacia de las intervenciones en salud bucal** | **2** |
| **9** | **Establecer grupos de trabajo. A nivel nacional para realizar capacitaciones y calibraciones sobre diagnóstico de enfermedades y patologías bucales** | **NO** | **Capacitación** |  |
| **10** | **Utilizar estrategias de comunicación virtuales para realizar estas capacitaciones.** | **NO** | **Capacitación** |  |
| **11** | **Establecer el número de odontólogos en el primer nivel de atención** | **SI** | **Magnitud, distribución y brecha de recursos humanos odontológicos** | **1** |
| **12** | **Capacitación en los centros educativos con el uso de las TICS** | **NO** | **Capacitación** |  |
| **13** | **Apoyarnos con Apps para capacitar a los profesionales primarios de salud** | **NO** | **Capacitación** |  |
| **14** | **Priorizar Cobertura Universal de Salud y generar Instituto Nacional de Salud Bucal que trabaje principalmente con el primer nivel del cuidado de salud** | **NO** | **Políticas** |  |
| **15** | **Padronizar el uso de herramientas para conocer el impacto de las intervenciones** | **NO** | **Políticas** |  |
| **16** | **Creación de aplicativos de prevención (apps)** | **SI** | **Desarrollo de TICS para actividades de prevención, promoción y control de enfermedades de salud bucal** | **2** |
| **17** | **El rol de la enseñanza Universitaria en pacientes CD, Capacitación en enseñanza de PCD, cuantos odontólogos atienden PCD** | **NO** | **Capacitación** |  |
| **18** | **Cartillas y material difusión virtual en prevención en SB** | **NO** | **Capacitación** |  |
| **19** | **Desarrollo de un instituto especializado de investigación craneofacial y dental que promueva la salud bucal y apoye la investigación a nivel de los trastornos y enfermedades del macizo craneofacial** | **NO** | **Proyecto de inversión** |  |
| **20** | **Terminología odontológica consensuada en la práctica privada y pública** | **NO** | **Capacitación** |  |
| **21** | **Uso de la telemedicina en la prevención y promoción de la salud oral** | **SI** | **Desarrollo de TICS para actividades de prevención, promoción y control de enfermedades de salud bucal** | **2** |
| **22** | **Uso de plataformas digitales como aplicaciones móviles para promover la prevención y promoción de la salud oral en la población** | **SI** | **Desarrollo de TICS para actividades de prevención, promoción y control de enfermedades de salud bucal** | **2** |
| **23** | **Creación de oficina de epidemiologia y/o inteligencia sanitaria especifica en patologías de salud bucal** | **NO** | **Gestión** |  |
| **24** | **Aplicación de las TICS para la creación de prevención de patologías pulpares y periapicales** | **SI** | **Desarrollo de TICS para actividades de prevención, promoción y control de enfermedades de salud bucal** | **2** |
| **25** | **Prevalencia de complicaciones post quirúrgicas (exodoncias) del cirujano dentista y del especialista en cirugía bucal y maxilofacial** | **SI** | **Estudio de prevalencia de complicaciones post quirúrgicas** | **1** |
| **26** | **Comparación entre radiografía convencional vs tomografía cone beam en la precisión del diagnóstico temprano de patología de los maxilares** | **SI** | **Estudio de métodos de identificación y/o diagnóstico odontológico** | **1** |
| **27** | **Determinar la demanda de especialistas en estomatología en las IPRESS Nivel II-III a nivel nacional.** | **SI** | **Magnitud, distribución y brecha de recursos humanos odontológicos** | **3** |
| **28** | **Determinar la eficiencia el en diagnóstico de las patologías del sistema estomatognático en cirujanos dentistas que laboran en IPRESS nivel I, II y III y su adecuado registro en el sistema epidemiológico.** | **SI** | **Estudios de calidad de atención en los servicios de atención odontológica en Establecimientos de salud** | **3** |
| **29** | **Niveles de satisfacción en los procedimientos de diagnóstico y tratamiento por patología del sistema estomatognático en pacientes en los niveles de atención I, II y III.** | **SI** | **Estudios de calidad de atención en los servicios de atención odontológica en Establecimientos de salud** | **3** |
| **30** | **Estudio sobre la necesidad de la atención odontológica por especialidades** | **SI** | **Estudios de requerimientos de servicios odontológicos especializados de la población peruana** | **3** |
| **31** | **Estudio sobre la necesidad de un sistema de Triaje para que pueda derivar al paciente al nivel de atención correspondiente.** | **NO** | **Práctica Clínica** |  |
| **32** | **Estudiar los servicios especializados de atención en el 2do y 3er nivel** | **SI** | **Magnitud, distribución y brecha de recursos humanos odontológicos** | **3** |
| **33** | **Estudiar el número de Centro Hospitalarios Estatales que disponen de centros de estudio del sueño multidisciplinarios.** | **NO** | **Planteamiento de Tema específico** |  |
| **34** | **adecuación de innovación de materiales y estrategias por regiones y por grupo etario** | **SI** | **Estudios de Evaluación de Tecnologías Sanitarias** | **3** |
| **35** | **Vigilancia epidemiológica de enfermedades bucales por medio de TICs** | **SI** | **Desarrollar estudios epidemiológicos en salud bucal** | **1** |
| **36** | **educación y evaluación de los cirujanos dentistas** | **NO** | **Capacitación** |  |
| **37** | **Desarrollo de plataformas digitales para compartir información actualizada para el cirujano dentista** | **NO** | **Capacitación** |  |
| **38** | **Innovación en la prevención y tratamiento de la caries dental** | **SI** | **Desarrollo de TICS para actividades de prevención, promoción y control de enfermedades de salud bucal** | **2** |
| **39** | **Innovación en la prevención y tratamiento de las maloclusiones** | **SI** | **Desarrollo de TICS para actividades de prevención, promoción y control de enfermedades de salud bucal** | **2** |
| **40** | **que tiempo es la demora para obtener el material o implementación a emplear diferentes TICS** | **NO** | **No es una necesidad de investigación per se** |  |
| **41** | **conocer si la población tiene acceso a diferentes medios de propagación de Tics** | **NO** | **Capacitación** |  |
| **42** | **Realizar un programa de información de los factores causantes de las diferentes maloclusiones dentarias** | **SI** | **Desarrollo y evaluación de programas de promoción** | **2** |
| **43** | **Facilitar información sobre los tratamientos alternativos de las diversas maloclusiones en las diferentes etapas de la vida** | **NO** | **Práctica Clínica - difusión** |  |
| **44** | **Evaluación de programas de promoción de salud bucal masiva en la población infantil, materna y geriátrica.** | **SI** | **Desarrollo y evaluación de programas de promoción** | **2** |
| **45** | **Investigar la aplicación de programas televisivos y de redes sociales para la promoción y prevención de caries bucal.** | **SI** | **Desarrollo y evaluación de programas de promoción** | **2** |
| **46** | **Uniformización de criterios para diagnosticar la enfermedad periodontal y periimplantaria** | **NO** | **Capacitación** |  |
| **47** | **Digitalización de historias clínicas, odontogramas y Periodontograma como mecanismos de diagnóstico y control** | **NO** | **Gestión** |  |
| **48** | **Teleperiodoncia e interdisciplinariedad con otras enfermedades crónicas no trasmisible** | **SI** | **Estudios que asocien enfermedades sistémicas y la salud bucal** | **1** |
| **49** | **Exámenes complementarios para el diagnóstico temprano de periodontitis** | **NO** | **Práctica Clínica** |  |
| **50** | **TICs en la prevención de enfermedades periodontales y periimplantarias** | **SI** | **Desarrollo de TICS para actividades de prevención, promoción y control de enfermedades de salud bucal** | **2** |
| **51** | **Innovación en la prevención, control y tratamiento de las enfermedades periodontales y periimplantarias** | **SI** | **Desarrollo y evaluación de programas de promoción** | **2** |
| **52** | **Resumir y compartir guías clínicas de diagnóstico y tratamiento de enfermedades crónicas no transmisibles entre el personal de salud, con el fin de poder realizar diagnósticos bidireccionales de enfermedad o necesidad de tratamiento, por parte de médicos y odontólogos, y así poder derivar oportunamente pacientes a diagnóstico y tratamiento periodontal y a diagnóstico y tratamiento de otras enfermedades crónicas no transmisibles.** | **NO** | **Capacitación** |  |
| **53** | **Facilitar la indicación y evaluación de exámenes auxiliares a odontólogos y capacitarlos en toma de muestras de saliva y fluido crevicular gingival. Los niveles de proteína C reactiva, interleucina 6, factor reumático y otros factores de riesgo para enfermedades crónicas no transmisibles, bajo peso al nacer y COVID-19 pueden ser detectados en muestras tomadas de la boca.** | **NO** | **Práctica clínica** |  |
| **54** | **Determinar el impacto de la educación sanitaria en salud bucal, según medio de difusión (redes sociales, entre otras).** | **SI** | **Evaluación de impacto** | **3** |
| **55** | **Determinar la calidad en la atención de la salud bucal y el impacto de procesos de mejora continua en la gestión de la misma.** | **SI** | **Estudios de calidad de atención en los servicios de atención odontológica en Establecimientos de salud** | **3** |
| **56** | **incorporar salud oral a salud general. difusión de las medidas de higiene a nivel masivo** | **SI** | **Desarrollo y evaluación de programas de promoción** | **2** |
| **57** | **Intervenciones a nivel comunitario, acudir a las poblaciones e implementar promoción de salud con hábitos comunes.** | **SI** | **Estudios que evalúen la eficacia de las intervenciones en salud bucal** | **2** |
| **58** | **Realizar programas de capacitaciones a cirujanos dentistas sobre estrategias de tratamiento para la enfermedad periodontal.** | **NO** | **Capacitación** |  |
| **59** | **Realizar una guía de manejo clínico para el tratamiento de enfermedades periodontales según el grupo etario y enfermedades sistémicas relacionadas.** | **NO** | **Práctica Clínica** |  |
| **60** | **Evaluación de la calidad de los registros de salud bucal en el Sistema de Información en Salud (HIS)** | **SI** | **Estudios de calidad de atención en los servicios de atención odontológica en Establecimientos de Salud** | **3** |
| **61** | **Evaluación de brecha de recurso humano odontológico.** | **SI** | **Magnitud, distribución y brecha de recursos humanos odontológicos** | **3** |
| **62** | **Estudios sobre la alfabetización en salud oral** | **SI** | **Desarrollo y evaluación de programas de promoción** | **2** |
| **63** | **Estudio sobre el uso de TICs para el acercamiento de la población a las intervenciones de salud bucal** | **SI** | **Desarrollo de TICS para actividades de prevención, promoción y control de enfermedades de salud bucal** | **2** |
| **64** | **Elaboración y/o validación de instrumentos, pruebas de diagnóstico y tamizaje de las patologías del sistema estomatognático más prevalentes** | **SI** | **Estudio de métodos de identificación y/o diagnóstico odontológico** | **1** |
| **65** | **Implementación de las TICS en la Capacitación del personal asistencial** | **NO** | **Capacitación** |  |
| **66** | **Impacto de la educación virtual en la salud bucal, creación de un programa educativo que acerque a los niños, adultos, adultos mayores y gestantes al uso correcto del cepillado dental, nutrición y autoevaluación de su salud bucal.** | **SI** | **Desarrollo de TICS para actividades de prevención, promoción y control de enfermedades de salud bucal** | **2** |
| **67** | **Aplicación de las diferencias morfológicas de la cabeza para la identificación de las personas (radiografía cefalometría, panorámica, periapical) reconstrucción facial forense (Cone Beam) erupción dentaria en la población peruana utilizando medios imagenológicos** | **SI** | **Estudio de métodos de identificación y/o diagnóstico odontológico** | **1** |
| **68** | **Crear una red de registro nacional de cáncer oral** | **NO** | **Ya existe HIS (CIE-10)** |  |
| **69** | **Capacitación a odontólogos generales del país, en detección de lesiones orales** | **NO** | **Capacitación** |  |
| **70** | **Educación sobre salud bucal** | **SI** | **Desarrollo y evaluación de programas de promoción** | **2** |
| **71** | **Uso racional de radiaciones en consultorio odontológico** | **NO** | **Capacitación** |  |
| **72** | **investigación en uso de herramientas tecnológicas y su aplicación en odontología** | **SI** | **Desarrollo de TICS para actividades de prevención, promoción y control de enfermedades de salud bucal** | **2** |
| **73** | **Establecer sistemas de TICs para la prevención de enfermedades bucales por etapas de vida** | **SI** | **Desarrollo de TICS para actividades de prevención, promoción y control de enfermedades de salud bucal** | **2** |
| **74** | **Interrelacionar programas nacionales de ayuda social y aseguramiento universal a la estrategia de salud bucal (plan esperanza, plan vacunación, nutrición para escolares etc.)** | **NO** | **Gestión** |  |
| **75** | **El rol de la enseñanza Universitaria en pacientes CD, Capacitación en enseñanza de PCD, cuantos odontólogos atienden PCD** | **NO** | **Capacitación** |  |
| **76** | **Cartillas y material difusión virtual en prevención en SB** | **NO** | **Capacitación** |  |
