# Supplementary material for: National priorities for oral health research, Peru 2022-2026: process, experiences and perspectives
Source: Rev Peru Med Exp Salud Publica. 2023 Sep 28;40(3):354–63. doi: 10.17843/rpmesp.2023.403.12082 (PMC10953641; doi:10.17843/rpmesp.2023.403.12082)
Supplement: Translation

# Translation: Prioridades nacionales de investigación en salud bucal, Perú 2022-2026: proceso, experiencias y perspectivas

**Authors:** Adriana Echevarria-Goche, Gilmer Solis-Sánchez, Lesly V. Tuesta-Orbe, Christian Andamayo-Flores, Margot Vidal-Anzardo

**Affiliations:** 1 Instituto Nacional de Salud, Lima, Perú.; 2 Ministerio de Salud, Lima, Perú.

**Keywords:** Prioridades de Investigación, Investigación en Sistemas de Salud Pública, Investigación Participativa Basada en la Comunidad, Política Nacional de Salud, Política de Investigación en Salud, Prioridades en Salud, Investigación Dental, Salud Bucal, Perú

## Abstract

El Instituto Nacional de Salud, ha llevado a cabo por primera vez el proceso de identificación de Prioridades Nacionales de Investigación en Salud Bucal a cargo de la Subdirección de Investigación y Laboratorios de Enfermedades No Transmisibles del Centro Nacional de Salud Pública con la asesoría técnica de la Dirección de Investigación e Innovación en Salud y en coordinación con la Dirección Ejecutiva de Salud Bucal de la Dirección General de Intervenciones Estratégicas en Salud Pública del Ministerio de Salud mediante metodología participativa de tres actores claves: investigadores/especialistas, expertos y decisores. El objetivo de este artículo es describir el proceso seguido para la identificación de estas prioridades, el cual comprendió cinco fases: i) identificación de objetivos estratégicos del MINSA, ii) identificación de necesidades de investigación en salud bucal, iii) revisión por expertos y valoración de las necesidades según criterios, iv) priorización (valoración de la lista de prioridades según calificación) y v) presentación de las prioridades. Como resultado se obtuvieron las 12 prioridades que posteriormente fueron aprobadas por Resolución Ministerial N.° 262-2022/MINSA, con un periodo de vigencia 2022-2026. Además, se brindaron recomendaciones para futuros procesos.

## INTRODUCCIÓN

El artículo 14 de la Constitución Política del Perú instaura como deber del Estado «promover el desarrollo científico y tecnológico del país» ^1^, y en esa misma línea, el numeral XV del Título Preliminar de la Ley N.º 26842, Ley General de Salud, establece que «el Estado promueve la investigación científica y tecnológica en el campo de la salud, así como la formación, capacitación y entrenamiento de recursos humanos para el cuidado de la salud» ^2^; de lo que se interpreta que el avance de la ciencia proviene de la educación y la investigación, las cuales son pilares para el desarrollo de la sociedad y el Estado garantiza su promoción.

El Ministerio de Salud (MINSA), como organismo del poder ejecutivo ^3^ tiene la misión de «proteger la dignidad personal promoviendo la salud, previniendo las enfermedades y garantizando la atención integral de salud de todos los habitantes del país, proponiendo y conduciendo los lineamientos de las políticas sanitarias en concertación con todos los sectores públicos y los actores sociales» ^4^. A partir del 2013 con la publicación del Decreto Legislativo N.° 1161, que aprueba la Ley de Organización y Funciones del MINSA, se establece la incorporación de la investigación y tecnología en salud bajo el ámbito de su competencia ^5^.

Asimismo, el Instituto Nacional de Salud (INS) tiene como visión, «ser una institución moderna, dinámica y líder a nivel nacional e internacional en la generación, desarrollo y transferencia de tecnologías y conocimientos científicos en investigación biomédica […]» ^6^. En ese marco, es que el INS lidera el desarrollo del proceso para la identificación de prioridades de investigación a nivel nacional, con el objetivo de generar el conocimiento en el campo de la salud pública a fin de orientar la formulación, implementación y evaluación de las políticas e intervenciones que destinen los recursos para la atención de problemas sanitarios priorizados debido a que los recursos en nuestro país son limitados ^7^.

La finalidad del presente artículo es describir el proceso seguido por la Subdirección de Investigación y Laboratorios de Enfermedades No Transmisibles (SUDENT) del Centro Nacional de Salud Pública (CNSP), con la asesoría técnica de la Dirección de Investigación e Innovación en Salud (DIIS) y en coordinación con la Dirección Ejecutiva de Salud Bucal de la Dirección General de Intervenciones Estratégicas en Salud Pública (DGIESP) del MINSA, para la identificación de las prioridades en investigación Prioridades Nacionales de Investigación en Salud Bucal; así como brindar recomendaciones para futuros procesos.

## PROCESOS PREVIOS

Históricamente en el Perú, se han desarrollado experiencias para identificar las prioridades de investigación en salud desde 1974, cada una de ellas ha guiado las políticas sanitarias desde entonces. En la primera, el MINSA expresó la intención de apoyar a los institutos de salud para las investigaciones; en la segunda (1983-1984) se desarrolló el Plan Nacional de Acción para alcanzar «salud para todos en el año 2000»; en la tercera (1991-1994), el Consejo Nacional de Ciencia, Tecnología e Innovación (CONCYTEC) y la Organización Panamericana de la Salud (OPS) trabajaron cuatro temas prioritarios y canalizaron recursos disponibles; en la cuarta (1996-1997) la Comisión de Políticas de Investigación en Salud del MINSA identificó nuevas áreas temáticas elaborando 234 fichas de estudios; en la quinta (2001-2002) el INS define los lineamientos de investigación a través de un seminario-taller interno; y durante los años 2006-2007 en su sexta edición, según Cabezas *et al*. ^8^, citado por la Dirección de Investigación e Innovación en Salud ^9^, «el INS profundiza el alcance y la metodología de establecer prioridades de investigación» consolidando su metodología participativa.

En el 2009, se desarrolló por primera vez el proceso de identificación de prioridades regionales que establecieron las prioridades para el periodo 2010-2014 definiéndose en esa oportunidad a la «investigación en salud» como: «la producción de conocimiento que tiene por objeto el estudio de las condiciones de salud (en el nivel biológico, psicológico y social) de un individuo o población y de las respuestas de la sociedad para mejorarlas» ^8^.

Para el 2014, se continúa el proceso comenzado en el 2009 con el desarrollo de talleres en las 24 regiones del Perú y Lima Metropolitana contando con la participación de autoridades regionales de salud, universidades públicas y privadas, entidades de investigación públicas y privadas, y otros sectores gubernamentales regionales convocados por el INS para establecer las Prioridades Nacionales de Investigación 2016-2021^7^^,^^9^, las cuales se vieron concretadas en el 2019, a través de la Resolución Ministerial N.° 658-2019/MINSA, que aprueba las Prioridades Nacionales de Investigación en Salud en Perú 2019-2023, identificándose los siguientes problemas sanitarios: i) Accidentes de tránsito; ii) Cáncer; iii) Enfermedades metabólicas y cardiovasculares; iv) Infecciones respiratorias y neumonía; v) Infecciones de transmisión sexual y VIH-SIDA; vi) Malnutrición y anemia; vii) Enfermedades metaxénicas y zoonóticas; viii) Salud ambiental y ocupacional; ix) Salud materna, perinatal y neonatal; y x) Salud mental ^10^. Es importante mencionar que tal como consta en el documento del proceso de identificación de dichas Prioridades Nacionales de Investigación en Salud, uno de los problemas sanitarios identificados fue la «salud bucal» ^11^; sin embargo, dado que se priorizaron los problemas que obtuvieron mayor puntaje, la salud bucal no figuraba en la lista de priorización final.

En este escenario, la salud bucal requiere una revalorización que le permita una mayor atención en cuanto a la investigación en nuestro país luego de haber sido relegada durante décadas, justificando una identificación específica de las Prioridades de Investigación en Salud Bucal.

En el 2021, el Instituto Nacional de Salud, a través de la SUDENT del CNSP, incorporó a la salud bucal como área de trabajo, estableciendo el «Plan de Salud Bucal 2021-2023», que inició con la identificación de Prioridades Nacionales de Investigación de Salud Bucal 2021-2025, y cuyo proceso será narrado en este artículo ^12^. Esta priorización será fundamental para guiar el desarrollo de investigaciones costo-efectivas y con el cual se visiona la optimización del uso de los recursos económicos del país tomando como referencia el proceso previo ^7^.

## EL PROCESO

La identificación de las Prioridades de Investigación en Salud Bucal que se desarrolló del 2 al 25 de junio del 2021 fue liderada por el equipo técnico de la SUDENT conformado por cuatro profesionales (un médico y tres cirujanos dentistas) quienes contaban con experiencia en investigación y formación académica en campos afines a Salud Pública, Epidemiología y/o Gestión de Servicios de Salud; asimismo, se contó con la asesoría del equipo técnico de la DIIS (oficina que ha liderado los procesos de Prioridades Nacionales de Investigación y ha validado la metodología durante la aplicación de este proceso de priorización de investigación), y trabajada en coordinación estrecha con el equipo técnico de la Dirección Ejecutiva de Salud Bucal (DSABU) de la DGIESP del MINSA. Las actividades se desarrollaron a través de talleres virtuales vía plataforma Zoom en base a metodología participativa adoptada de la «Guía para la identificación de Prioridades Regionales de Investigación en Salud» elaborada por la DIIS del INS ^9^^,^^13^.

Se establecieron tres actores claves considerando las siguientes características (Tabla 1):

 1. Investigadores/especialistas, conformado por aquellos que cumplían al menos dos de las cuatro condiciones: i) ser investigador reconocido por el Registro Nacional Científico, Tecnológico y de Innovación Tecnológica (RENACYT) (vía plataforma de DINA-CONCYTEC ^14^ a la fecha de descarga al 30 de marzo del 2021; ii) haber publicado dos o más artículos de investigación con relevancia en salud bucal (no se consideraron cartas al editor) en revistas por pares nacionales o internacionales; y posteriormente también se consideró: iii) ejercer docencia en universidad pública o privada; y iv) ser especialista en un área odontológica. Del listado de profesionales identificados que cumplían con las condiciones señaladas, el equipo técnico de la SUDENT logró tener acceso al número de teléfono y/o correo electrónico de 78 profesionales, medios que fueron empleados para establecer contacto mediante redes sociales, llamadas telefónicas y/o WhatsApp con la finalidad de convocarlos a participar del proceso; de los 78 profesionales contactados, se contó con la respuesta y aceptación de 46 (59,0%). Esta convocatoria fue realizada entre el 18 y el 28 de mayo del 2021.

**Tabla 1:** Participantes del proceso de las Prioridades Nacionales de Investigación en Salud Bucal.

Fases	Objetivos	Investigadores/ especialistas n=46	Decisores n=32	Expertos ^a^ n=7	Total
1	Identificación de los objetivos estratégicos del MINSA	-	2 ^b^	-	2
2	Identificación de necesidades de investigación en salud bucal	39	-	-	39
3	Revisión por expertos y valoración de las necesidades según criterios				
3a	Revisión de las necesidades por expertos	-	3 ^b^	3	6
3b	Valoración de las necesidades según criterios	28	14	1	43
4	Priorización (valoración de la lista de prioridades según calificación)	-	3 ^b^	-	3
5	Presentación de las prioridades	11	29 ^c^	1	41

a Expertos conformados por la Resolución Ministerial N.° 571-2017/MINSA.

b Miembros de la DSABU/MINSA.

c Pertenecientes a 15 regiones.

2. Decisores, conformado por los tres integrantes del equipo de la Dirección Ejecutiva de Salud Bucal (por disponibilidad en algunas fases intervinieron 2) y los 29 Coordinadores del programa de salud bucal pertenecientes a las Direcciones Regionales de Salud (DIRESAS), Gerencias Regionales de Salud (GERESAS) y Dirección de Redes Integradas de Salud (DIRIS). El listado de los coordinadores del programa nacional salud bucal fue brindado por la DSABU, entidad que brindó el apoyo logístico para gestionar la convocatoria vía correo electrónico; dicha convocatoria se realizó entre 18 y el 28 de mayo de 2021, contando con la respuesta y aceptación de todos los coordinadores, considerando un total de 32 decisores.

3. Expertos en Salud Bucal, conformados por Comité de Expertos *ad honorem* de la DSABU de la DGIESP mediante Resolución Ministerial N.° 571-2017/MINSA ^15^. DSABU proporcionó los contactos de los siete integrantes del referido comité, a quienes se les convocó mediante llamadas telefónicas y/o WhatsApp entre 18 y el 28 de mayo del 2021. Luego de la convocatoria se contó con la respuesta y aceptación de tres expertos (42,9%). 

A continuación, se describen las fases del proceso:

### Fase 1. Identificación de objetivos estratégicos del MINSA

No existiendo un plan Nacional de Salud Bucal que tuviera objetivos estratégicos planteados, éstos fueron identificados por los miembros de la DSABU en base a los lineamientos de las políticas vigentes del MINSA, con la orientación metodológica del equipo del INS como se señala en la Tabla 2.

**Tabla 2:** Objetivos estratégicos para la identificación y determinación de Prioridades de Investigación en Salud Bucal 2021-2025.

#	Objetivos estratégicos
1	Desarrollar un sistema epidemiológico de morbilidad y factores asociados en salud bucal, para sustento normativo y toma de decisiones según curso de vida
2	Establecer intervenciones para la prevención, control, tratamiento de patologías bucales enfocado en la persona, familia y comunidad que impactan en la calidad de vida
3	Fortalecer los procesos de gestión para la implementación de acciones de mejora en las intervenciones de salud bucal

### Fase 2. Identificación de necesidades de investigación en salud bucal

Esta fase fue ejecutada en dos etapas:

*Primera Etapa. Identificación inicial*

En esta etapa participaron 39 de los 46 investigadores/especialistas (84,8%), señalados en los actores clave, quienes elaboraron la formulación inicial de necesidades de investigación; cabe precisar que entre los 39 participantes se cubrió la totalidad de especialidades odontológicas ^16^, con lo cual se contó con propuestas desde diferentes posiciones, las que se complementaron entre sí. Como resultado de esta etapa se obtuvo un total de 220 necesidades de investigación propuestas (Material suplementario).

*Segunda Etapa. Clasificación y agrupamiento*

En esta etapa, los cuatro integrantes del equipo de la SUDENT realizaron todos los procedimientos bajo consenso, como la estandarización de la redacción de las propuestas iniciales, en base de cinco tipos de investigación (a. Investigaciones para medición de la magnitud y distribución de problemas de salud, b. Investigación para el entendimiento de diversas causas o determinantes del problema de salud, c. Investigaciones de desarrollo de soluciones o intervenciones que ayuden a prevenir y/o mitigar problemas de salud, d. Investigaciones para realizar el traslado de las soluciones o evidencia en políticas prácticas y productos, e. Investigaciones de evaluación del impacto de soluciones o intervenciones) ^9^^,^^17^; otrosí reclasificaron las 220 necesidades según objetivos estratégicos al cual realmente pertenecían y depuró las duplicadas. También se reformuló la redacción de dichas necesidades, obteniendo así un segundo listado de 32 que posteriormente fueron agrupadas en 18 necesidades propuestas (Material suplementario).

### Fase 3. Revisión por expertos y valoración de las necesidades según criterio

Realizada en dos etapas:

*Primera Etapa. Revisión de las necesidades por expertos*

Los tres expertos revisaron y plantearon adecuaciones en la redacción de algunas de las necesidades propuestas, e incorporaron un par de necesidades por ser de impacto en la salud pública: «i) Desarrollar estudios que evalúen el impacto de enfermedades emergentes/reemergentes relacionados a la salud bucal; y ii) Estudios de prevalencia de complicaciones relacionadas a recepción o ausencia de tratamiento». El producto obtenido fue el listado definitivo de las necesidades de investigación en salud bucal.

*Segunda etapa. Valoración de las necesidades según criterios.*

Una vez obtenida la lista de 20 necesidades de investigación, ésta fue calificada por los participantes distribuidos en los tres grupos de actores claves, debido a aspectos de disponibilidad de los actores, no se pudo contar con la totalidad de ellos (Tabla 1). Dicha calificación fue realizada considerando los criterios y escala de valoración planteada en la Guía Metodológica de la DIIS (Tabla 3), los puntajes obtenidos (Tabla 4) permitieron generar un listado ordinal descendente independiente de las necesidades de investigación por cada objetivo estratégico.

**Tabla 3:** Criterios y escala de valoración para la calificación de las Prioridades Nacionales de Investigación en Salud ^9^^,^^17^.

Criterios	Escala de valoración
Brecha de conocimiento: necesidad de generar conocimiento para acortar la distancia entre la situación actual y lo que debería ser, se considera brecha cuanto menos estudiado se encuentra el tema	1 = muy bajo 2 = bajo 3 = medio 4 = alto 5 = muy alto
Factibilidad: posibilidad de investigar desde el punto de vista político, técnico, financiero y operativo (disponibilidad de recursos necesarios, y/o se puede acceder a ellos a través de convenios, alianzas u otros mecanismos)	
Efectos o consecuencias: impacto que se obtendrían para la población en general y para el sistema de salud	

**Tabla 4:** Sumatoria de Calificación de las Prioridades Nacionales de Investigación en Salud Bucal por objetivo estratégico.

#	Objetivos estratégicos	#	Necesidades de investigación	Sumatoria de calificación
1	Desarrollar un sistema epidemiológico de morbilidad y factores asociados en Salud Bucal, para sustento normativo y toma de decisiones según curso de vida.	1	Desarrollar estudios epidemiológicos descriptivos de las condiciones y enfermedades del sistema estomatognático por curso de vida.	535
		2	Estudios para identificar determinantes y factores asociados a patologías bucales según curso de vida.	504
		3	Estudios que asocien enfermedades sistémicas y no transmisibles con la prevalencia e incidencia de patologías bucales.	504
		4	Estudios de prevalencia de complicaciones relacionadas a recepción o ausencia de tratamiento odontológico.	491
2	Establecer intervenciones para la prevención, control, tratamiento y mantenimiento de patologías bucales que impactan en la calidad de vida, enfocado en la persona, familia y comunidad.	1	Desarrollo de Innovaciones y/o Tecnologías de Información y Comunicación (TIC) para las intervenciones en salud bucal (prevención de enfermedades del sistema estomatognático, promoción, recuperación y rehabilitación).	541
		2	Desarrollo y evaluación de programas de prevención y promoción en salud bucal por curso de vida.	528
		3	Desarrollo y evaluación de programas para la salud bucal en población vulnerable.	528
		4	Estudios de seguimiento y adherencia a intervenciones relacionadas a salud bucal.	519
		5	Desarrollar y evaluar la eficacia de intervenciones relacionadas a salud bucal.	518
		6	Estudios de evaluación del impacto de la salud sistémica luego del tratamiento odontológico.	518
		7	Estudios de métodos de identificación y diagnóstico odontológico.	483
		8	Estudios experimentales *in vitro* y/o ensayos clínicos relacionados a salud bucal.	425
3	Fortalecer los procesos de gestión para la implementación de acciones de mejora en las intervenciones de salud bucal.	1	Magnitud, distribución y brecha de recursos humanos odontológicos.	535
		2	Estudios de evaluación de impacto de las enfermedades bucales por curso de vida.	534
		3	Estudios del impacto económico y social del acceso, oportunidad y eficacia de tratamientos odontológicos.	516
		4	Estudios de Evaluación de Tecnologías Sanitarias en salud bucal.	510
		5	Estudios de evaluación económica en salud bucal.	500
		6	Desarrollar estudios que evalúen el impacto de enfermedades emergentes/reemergentes relacionadas a la salud bucal.	498
		7	Desarrollar estudios de análisis de oferta y demanda de los servicios odontológicos.	491
		8	Estudios de calidad de atención en los servicios de atención odontológica en establecimientos de salud.	490

### Fase 4. Priorización (valoración de la lista de prioridades según calificación)

En esta fase participaron el equipo técnico de la SUDENT y la DSABU, quienes, por consenso, luego de revisar las calificaciones de las necesidades (Tabla 4), decidieron realizar modificaciones que afectaron el objetivo 2:

Eliminar la necesidad 3 (desarrollo y evaluación de programas para la salud bucal en población vulnerable) porque se encontraba contenida en la necesidad 2 de dicho objetivo (desarrollo y evaluación de programas de prevención y promoción en salud bucal por curso de vida).

Reagrupar la necesidad de investigación 5 (desarrollar y evaluar la eficacia de intervenciones relacionadas a salud bucal) y 8 (estudios experimentales *in vitro* y/o ensayos clínicos relacionados a salud bucal), la cual fue valorada en último lugar (Tabla 4); sin embargo, fueron consideradas para la reagrupación dada su re­levancia y nivel metodológico, por lo que finalmente la redacción fue «desarrollar estudios experimentales y/o de evaluación de eficacia de intervenciones relacionadas a salud bucal».

Tras las modificaciones planteadas, se seleccionaron las cuatro primeras necesidades de investigación por cada objetivo, obteniendo así el listado final de 12 prioridades de investigación (Tabla 5).

**Tabla 5:** Prioridades Nacionales de Investigación en Salud Bucal 2022-2026 aprobadas por Resolución Ministerial N.° 262-2022/MINSA.

Objetivo estratégico	Necesidades de investigación
Desarrollar un sistema epidemiológico de morbilidad y factores asociados en salud bucal, para sustento normativo y toma de decisiones según curso de vida.	Desarrollar estudios epidemiológicos descriptivos de las condiciones y enfermedades del sistema estomatognático por curso de vida.
	Estudios para identificar determinantes y factores asociados a patologías bucales según curso de vida.
	Estudios que asocien enfermedades sistémicas y no transmisibles con la prevalencia e incidencia de patologías bucales.
	Estudios de prevalencia de complicaciones relacionadas a recepción o ausencia de tratamiento odontológico.
Establecer intervenciones para la prevención, control, tratamiento y mantenimiento de patologías bucales que impactan en la calidad de vida, enfocado en la persona, familia y comunidad.	Desarrollo de Innovaciones y/o Tecnologías de Información y Comunicación (TIC) para las intervenciones en salud bucal (prevención de enfermedades del sistema estomatognático, promoción, recuperación y rehabilitación de la salud).
	Desarrollo y evaluación de programas de prevención y promoción en salud bucal por curso de vida.
	Estudios de seguimiento y adherencia a intervenciones relacionadas a salud bucal.
	Desarrollar estudios experimentales y/o de evaluación de eficacia de intervenciones relacionadas a salud bucal.
Fortalecer los procesos de gestión para la implementación de acciones de mejora en las intervenciones de salud bucal.	Magnitud, distribución y brecha de recursos humanos odontológicos.
	Estudios de evaluación de impacto de las enfermedades bucales por curso de vida.
	Estudios del impacto económico y social del acceso, oportunidad y eficacia de tratamientos odontológicos.
	Estudios de Evaluación de Tecnologías Sanitarias en salud bucal.

### Fase 5. Presentación de las prioridades

La SUDENT presentó a los actores claves del proceso (investigadores/especialistas, expertos y decisores) el listado final de las Prioridades Nacionales de Investigación en Salud Bucal; tras lo cual se realizaron los procedimientos administrativos bilaterales entre el INS y el MINSA para su aprobación, y posteriormente se oficializó mediante Resolución Ministerial N.° 262-2022/MINSA con fecha 30 de marzo de 2022 considerando una vigencia para el periodo 2022-2026 ^18^, con un horizonte de cinco años al igual que en los procesos previos ^10^^,^^19^^,^^20^. Durante el evento de presentación realizado el 25 de junio del 2021 no se pudo contar con la participación de la totalidad de actores por aspectos de disponibilidad horaria; sin embargo, en el caso de las regiones de Junín, Ica y Loreto, se contó con representantes conectados de manera adicional al coordinador de la estrategia de salud bucal, concurriendo así 29 participantes de 15 regiones (Tabla 1).

## RESULTADOS Y COMPARATIVAS

Las prioridades de Investigación son definidas desde el INS como «necesidades de investigación priorizadas en base a los problemas sanitarios que más afectan a la población y que requieren de respuestas efectivas eficientes» ^21^. Esta importante labor ha sido desarrollada por el INS en varias oportunidades, de cuales se han materializado en Resoluciones Ministeriales como la Resolución Ministerial N.° 262-2022/MINSA ^18^ que aprueba las «Prioridades Nacionales de Investigación en Salud Bucal» (Tabla 5), que son materia de este artículo.

El Instituto Nacional de Investigación Dental y Craneofacial de los Estados Unidos, conocido como NIDCR, ha elaborado su Plan Estratégico 2021-2026, basado en cuatro principios guía y cinco prioridades estratégicas con sus respectivos objetivos ^22^. De igual modo, el Instituto Nacional para la Investigación en Salud (NIHR) de Inglaterra ha establecido las 10 prioridades de salud bucodental ^23^. Al revisar los objetivos del plan y las preguntas identificadas por la comunidad inglesa, se encontró similitudes en algunas de ellas y las prioridades identificadas en Perú ^18^; siendo la principal diferencia de ambos procesos y el nuestro, los participantes. Este proceso se efectuó con la participación de investigadores/especialistas, decisores y expertos (no hubo participación de la sociedad civil); mientras que en EE.UU. participaron diversos organismos relacionados a la comunidad científica, agencias federales y la industria; y en Inglaterra participaron el grupo NIHR en colaboración con las escuelas de odontología y la salud pública de Inglaterra para identificar las preguntas no respondidas relacionadas a la investigación sobre salud bucodental de los pacientes, del público, cuidadores y profesionales de la salud bucal.

Por otra parte, en Irán, país en desarrollo como el nuestro, el estudio de identificación de prioridades del 2018, encontró 171 tópicos en seis áreas relacionadas a la Salud Bucal, obteniéndose en su proceso 44 tópicos sobre el «cuidado clínico dental y tratamiento», 37 tópicos en «salud y prevención», 19 tópicos en «promoción de la salud y educación», 37 tópicos en «manejo de la prestación de la atención dental», 16 tópicos en «políticas y sus requerimientos» y 18 tópicos en «materiales dentales, equipamiento y tecnologías» ^24^, tópicos que coinciden en su gran mayoría con los identificados en el Perú ^18^.

En Latinoamérica, Brasil consideró a la salud bucal en su Agenda Nacional de Prioridades de Investigación en Salud en el 2015, cuya subagenda, coincide con las Prioridades Nacionales de Investigación en Salud Bucal de Perú en los tópicos referentes a epidemiología, factores de riesgo, desarrollo del sistema epidemiológico, desarrollo de estrategias para la promoción de salud bucal, innovaciones para los tratamientos y oferta de servicios de salud bucal ^25^.

Las Prioridades de Investigación en Salud Bucal identificadas en Perú son afines a las prioridades de investigación para el fortalecimiento de la Acción de Salud Global para la Salud Bucal; las cuales, tienen su origen en las brechas de conocimiento en: i)Epidemiología y sistemas de información de salud para la vigilancia de las condiciones orales; ii)Colección, armonización y evaluación rigurosa de la evidencia para la equidad en la prevención y tratamiento de las condiciones orales; y iii)Estrategias para brindar calidad esencial en el cuidado de la salud bucal sin dificultades financieras ^26^.

## IMPACTO ESPERADO

La situación de la salud bucal en el Perú constituye un problema de salud pública, dado que ocupa el segundo lugar de las 10 principales causas de morbilidad en el Perú en las tres regiones naturales (costa, sierra y selva) ^27^ y la emergencia sanitaria ocasionada por la pandemia por el COVID-19, ha agravado la salud bucal de la población peruana, por las restricciones en el acceso a los servicios de salud, en especial el odontológico, adoptadas para mitigar los contagios ^28^^-^^31^ por lo que se requiere la identificación e implementación de nuevas intervenciones sanitarias, lineamientos y políticas públicas en este campo de la salud bucal teniendo en cuenta el contexto actual. Por tanto las «Prioridades Nacionales de Investigación en Salud Bucal 2022-2026» identificadas ^18^, tienen un rol trascendental para orientar las investigaciones que se realizarán en salud bucal y servirán a su vez para redefinir políticas y establecer y/o reformular programas ^32^ más efectivos que permitan contribuir al mejoramiento del sistema de salud ^33^, acorde a la Ley N.° 31540, que declara de interés nacional y necesidad pública la formulación y ejecución de la política nacional de salud bucal ^34^.

## LECCIONES APRENDIDAS, PERSPECTIVAS Y RECOMENDACIONES

Se debe resaltar además que en este proceso se han considerado de forma censal como actores a los decisores de la gestión pública en salud bucal tanto a nivel central como regional, quienes pueden tener una rotación o alta movilidad según cambios políticos; por lo que resultó importante contar con la visión de la academia, representado por profesionales que evidencian la realización de investigación.

Resulta relevante comprender el contexto social en el cual se llevó a cabo el proceso, toda vez que al existir algunas restricciones para la realización de coordinaciones presenciales producto de la pandemia por la COVID-19, todas las coordinaciones y actividades se realizaron de manera virtual; hecho que no garantizó contar con una participación total de los actores inicialmente considerados; en cambio, se logró tener participación de todos los actores en todas las etapas y fases del proceso, lo cual permitió tener participación desde todos los enfoques.

En cuanto a la conducción del proceso, la cual fue realizada por el equipo de la SUDENT, se debe destacar que todas las acciones realizadas que involucraron cambios en las propuestas obtenidas fueron realizadas bajo consenso y posteriormente revisadas en la etapa subsiguiente para ser aceptadas.

Tomando en cuenta que el proceso se condujo siguiendo los lineamientos establecidos en la Guía Metodológica de la DIIS ^9^, bajo la asesoría técnica de dicho órgano (el cual ha llevado a cabo procesos previos de identificación de prioridades nacionales de investigación en salud); no se contempló la realización de la prepublicación del listado de prioridades identificadas debido a que durante todo el proceso se recibió la crítica y recomendaciones de los actores clave invitados, de quienes además se valoraron todos los aportes y comentarios de forma abierta; sin embargo, cabe la posibilidad de que el haber omitido la prepublicación, así como la declaración de conflictos de interés, podrían ser consideradas como limitaciones.

Adicionalmente, otra limitación en la conducción del proceso estuvo relacionada a las dificultades de los actores claves para tener participación continua en todas las fases y actividades, lo cual estuvo relacionado a aspectos de disponibilidad horaria; sin embargo, debemos resaltar que dicha dificultad se mitigó en su mayor magnitud al conducir el proceso de manera virtual. En el sentido de la variabilidad en la tasa de participación, debemos reconocer un potencial sesgo de participación por voluntariedad, el cual podría representar una motivación que potencial no fuese declarada como conflicto de interés y que no pudiese ser identificada o advertida.

Se reconoce la existencia de posibles sesgos de selección, dado que si bien, se utilizó la relación de investigadores con código de RENACYT (30 investigadores según la fecha de descarga) para convocar a los actores claves del grupo que representa a los investigadores, y, aunque la mayoría puso a disposición su correo electrónico de contacto, no se obtuvo de la totalidad; razón por la cual se procedió a ubicarlos a través de red de contactos, por redes sociales y/o WhatsApp; no obstante, algunos no respondieron, o la línea estaba fuera de servicio, o no contaban con disponibilidad. Ante esta situación, se decidió incluir al proceso a cirujanos dentistas relacionados a la comunidad científica como docentes, quienes, como parte de su labor, dirigen el desarrollo de protocolos de investigación a través de los diferentes cursos impartidos en las universidades", y especialistas, quienes aplican los resultados de las investigaciones directamente en el paciente. Por consiguiente, se considera que la representatividad de la comunidad científica de salud bucal pudo verse afectada.

En conclusión, este artículo ha descrito el proceso seguido para la identificación por primera vez de la Prioridades de Investigación en Salud Bucal en el Perú, las cuales cuentan con una vigencia del 2022 al 2026, las mismas que orientarán el desarrollo de investigaciones que servirán de evidencia para la toma de decisiones en salud bucal, esperando además que el proceso descrito sirva de modelo para la identificación de un nuevo listado de prioridades de investigación en este campo, el cual pueda ser perfeccionado en cada nuevo proceso.

Luego de haberse llevado el proceso de identificación de las prioridades tomando en cuenta la Política Nacional Multisectorial de Salud al 2030 ^35^, el reporte publicado de la Salud Bucal Global realizada por la Organización Mundial de la Salud ratifica que la Salud Bucal requiere una acción urgente y visibiliza los desafíos y oportunidades hacia la salud bucal ^36^^)^ que debemos tener en cuenta para las propuestas de investigación alineadas con las prioridades identificadas en este proceso.

Convocamos a todos los profesionales investigadores de la salud bucal a contribuir con el desarrollo de la producción científica peruana a través de investigaciones que se enmarquen en dichas Prioridades, contribuyendo de esta manera con la evidencia científica puesta a disposición para la construcción de las políticas públicas sólidas en beneficio de la sociedad peruana. En adición, recomendamos a la DIIS evaluar la pertinencia de la incorporación de la prepublicación de los resultados y la declaración de conflictos de interés como parte de la metodología para futuros procesos.

De igual modo, será necesario impulsar la implementación de las Prioridades Nacionales de Investigación en Salud Bucal en la academia, para fomentar su desarrollo en las universidades de todo el país además, permitirá a los organismos competentes incluir el tema de salud bucal en los fondos concursables, para incentivar a los investigadores a desarrollar investigaciones que requieran mayor presupuesto como es el caso de los ensayos clínicos y las innovaciones tecnológicas, considerando la importancia del financiamiento para las investigaciones de la más alta calidad tomando como modelo los Institutos Nacionales de Salud ^37^. Las prioridades identificadas en cuanto a su planteamiento representan un nivel macro para la planificación y gestión de las investigaciones en salud bucal, las cuales deben servir para que tanto instituciones nacionales públicas y privadas, así como la academia, puedan formular sus agendas de tópicos/líneas/ideas de investigación las cuales de forma más específica permitan promover el planteamiento de preguntas de investigación que se alinean con las necesidades de generación de evidencia y garanticen un uso adecuado de los limitados recursos con los que se cuentan.
